# A Cloud-Assisted Random Linear Network Coding Medium Access Control Protocol for Healthcare Applications

**DOI:** 10.3390/s140304806

**Published:** 2014-03-10

**Authors:** Elli Kartsakli, Angelos Antonopoulos, Luis Alonso, Christos Verikoukis

**Affiliations:** 1 Department of Signal Theory and Communications (TSC), Technical University of Catalonia (UPC—BarcelonaTECH), C./ Esteve Terradas 7, C4-202P, Castelldefels 08860, Spain; E-Mail: luisg@tsc.upc.edu; 2 Telecommunications Technological Centre of Catalonia (CTTC), Castelldefels 08860, Spain; E-Mails: aantonopoulos@cttc.es (A.A.); cveri@cttc.es (C.V)

**Keywords:** MAC protocol design, network coding, cloud, WBANs, WSNs, healthcare

## Abstract

Relay sensor networks are often employed in end-to-end healthcare applications to facilitate the information flow between patient worn sensors and the medical data center. Medium access control (MAC) protocols, based on random linear network coding (RLNC), are a novel and suitable approach to efficiently handle data dissemination. However, several challenges arise, such as additional delays introduced by the intermediate relay nodes and decoding failures, due to channel errors. In this paper, we tackle these issues by adopting a cloud architecture where the set of relays is connected to a coordinating entity, called cloud manager. We propose a cloud-assisted RLNC-based MAC protocol (CLNC-MAC) and develop a mathematical model for the calculation of the key performance metrics, namely the system throughput, the mean completion time for data delivery and the energy efficiency. We show the importance of central coordination in fully exploiting the gain of RLNC under error-prone channels.

## 1. Introduction

The widespread use of information and communication technologies in today's medical and health services, usually described by the term eHealth, is drastically changing the face of healthcare delivery. More recently, the rapid advances in mobile and wireless communications offering almost ubiquitous and continuous connectivity through intelligent mobile devices, such as smartphones, tablets, personal digital assistants (PDAs), *etc.*, have added a new component to the eHealth paradigm, known as mHealth [1]. A broad range of eHealth/mHealth scenarios with benefits for both patients and healthcare providers are envisioned, including remote patient monitoring, active management of chronic diseases, such as diabetes, support for independent aging to the elderly and the tracking of personal fitness activities to improve health and well-being [2].

A number of small and autonomous medical sensor devices, either wearable or implantable, are usually employed at the patient's side. Each sensor is typically highly specialized to perform a specific task, such as collecting patient's vital signs (e.g., body temperature, brain activity, heart rate, *etc.*), measuring external parameters (e.g., ambient temperature, motion patterns, patient location, *etc.*) or even performing specific actions (e.g., the administration of a specific dosage of insulin). The sensors are usually connected to a sink central device that acts as a coordinator (*i.e.*, a collector of all the sensory data) through a short-range wireless technology, thus forming a wireless body area network (WBAN). The IEEE 802.15 Task Group 6 has recently issued a new standard, the IEEE 802.15.6 specification, specifically targeted at WBANs [3].

Apart from the local sensor interconnection handled by the WBAN, it is important to provide an end-to-end communication bridge between the patient and the healthcare provider. Depending on the application scenario, this task can be accomplished through different technologies, from long-range cellular 3G/4G systems to local deployments of wireless sensor networks (WSNs). The latter solution is very appealing when radio limitations are present, as, for example, in indoor environments with poor cellular coverage (e.g., a hospital ward). Recently, the deployment of ambient WSNs for healthcare applications is being considered in the literature. Ambient sensors are usually employed to enhance context-awareness, by providing relevant environmental information for the patient's surroundings [4,5]. However, it is also possible to use the deployed sensors as relays that forward the data collected by the WBAN to the medical server [6,7].

The design of efficient medium access control (MAC) protocols and routing techniques is crucial to handle the flow of information through the distribution WSN. The application of random linear network coding (RLNC) [8] over the MAC and network layers has been studied as an innovative way for data dissemination and forwarding, especially in relay networks. The main idea behind RLNC is to transmit linear combinations over a block of original data packets, created by multiplying each packet with a random coefficient drawn by a finite Galois field. For instance, a relay can forward a linear combination of its received packets. Thus, the destination does not need to acknowledge each packet individually, but instead, it has to receive a sufficient number of linearly-independent encoded packets to be able to extract the original information.

In the context of relay-assisted WBANs, RLNC techniques are often employed as a means to enhance reliability. The work presented in [9] shows the potential throughput improvement under error-prone channels through the application of RLNC, as a function of the number of redundancy packets, the employed relays and the number of sink nodes. More advanced schemes, such as cooperative diversity coding, where both coded and uncoded data packets are transmitted, can yield even better results [10]. A tree topology has been assumed in [11], where multiple nodes communicate with the coordinator through a small number of relays. The nodes send their uncoded data to the relays, which, in turn, forward a set of both coded and uncoded packets to the destination. The obtained results show that RLNC can offer higher reliability with respect to traditional redundancy schemes, where multiple retransmissions of uncoded packets take place. Similar conclusions are drawn in [12], where the coding decisions take into account the reliability requirements of different traffic flows.

Nevertheless, most schemes assume deterministic transmission sequences, without considering MAC layer channel access rules. In addition, their performance significantly drops when channel errors are introduced, since high redundancy is required in the sense that more retransmissions must take place in order for the receiver to successfully decode the received encoded packets. As a result, the potential RLNC gain is not fully exploited. These problems can be mitigated by employing a MAC protocol that enables the exchange of information among the relays. However, relay cooperation usually introduces significant overheads and complexity that may not always be supported by low-power WSNs or WBANs. An alternative approach is to adopt a centralized architecture, where all the relays form part of a cloud infrastructure that is controlled by a central entity.

In this paper, we focus on an ambient WSN that acts as a distribution network, connecting one or more WBANs (sources) to a central process unit (destination), and investigate MAC schemes that efficiently handle the flow of information between the two communication ends. Initially, as a reference scheme, we consider baseline MAC (BS-MAC) based on the IEEE 802.15.6 MAC mechanism with slight modifications to support the multi-hop relay topology. In continuation, we apply the RLNC principles to the baseline scenario (NC-MAC), to show the potential gains and to identify significant performance weaknesses in the presence of channel errors. Finally, we propose a cloud-assisted RLNC-based MAC (CLNC-MAC) that employs centralized control to coordinate transmissions in the relay network, in order to exploit the benefits of RLNC and enhance both performance and reliability.

Very few related works can be found in the literature in this context. In [13], a cloud-assisted MAC protocol has been proposed and implemented for wireless local area network (WLAN) deployment. The main idea is to transform the access points into a unified user interface and concentrate MAC layer functions and processing to virtual machines provided by cloud services. Even though the practical contribution of this work is significant, no enhancements are made with respect to the MAC layer mechanism. In particular, the main discussion focuses on implementation issues, *i.e.*, the modification of WLAN cards for virtual interconnection through an OpenFlow switch, whereas the IEEE 802.11 MAC is employed with no major modifications. The aim of our work is different, since we adopt the cloud architecture as a means to exploit the potential of RLNC in cooperative relay scenarios. We propose modifications on the IEEE 802.15.6 MAC layer protocol in order to coordinate the request for retransmissions and the data relaying with the help of the cloud, thus enhancing the decoding process of the NC packets at the destination. In [14], a relay cloud model has been proposed, in which a subset of relay nodes opportunistically forms a cloud that is enabled to transmit simultaneously, thus increasing the spatial diversity in the network. This approach is closer to our work, since a cloud-based cooperative MAC protocol is proposed and evaluated. However, there are two key differences with respect to our proposal: (i) RLNC is not considered, and relays transmit the same uncoded packets simultaneously, thus creating virtual multiple-input-multiple-output (MIMO) links; and (ii) the role of the relay cloud is fundamentally different. In particular, each relay in the cloud decides in a distributed way whether to participate in a cooperation phase based on a transmission probability. In our work, however, the cloud plays an active role in the exchange of information among the relays and the coordination of the transmissions, leading to a centralized scheme.

The contributions of our work are summarized as follows:

- We study different MAC mechanisms to handle the data flow in a distribution WSN. We explore the potential benefits of RLNC, but we also identify performance flaws under error-prone channels. As a solution, we propose CLNC-MAC, a novel cloud-assisted MAC protocol that employs RLNC and guarantees the successful delivery of all generated data to the destination through a relay network, regardless of the channel conditions.
- We develop an analytical framework for CLNC-MAC based on queuing theory for the calculation of key performance metrics, namely the average throughput, data delay and energy efficiency, and we validate the theoretical expressions through extensive Monte Carlo simulations.
- We provide a thorough performance assessment of the three MAC schemes (BS-MAC, NC-MAC and CLNC-MAC) in the presence of channel errors for a two-hop relay network, and we show the significant performance enhancements that can be achieved through the combination of RLNC with centralized control.

The remainder of this paper is organized into five sections. The application scenario and the system model are described in Section 2. In Section 3, we present the three considered MAC protocols, and in Section 4, we provide the mathematical analysis for the calculation of the key performance metrics of CLNC-MAC. The performance evaluation and the discussion of the obtained results follows in Section 5. Finally, Section 6 summarizes the main conclusions of this work.

## 2. Application Scenario

In this section, we describe the proposed healthcare application scenario. We first briefly present the considered end-to-end framework, and then, we focus on the system model employed for the design and evaluation of the proposed MAC schemes.

### 2.1. Framework of the End-to-End Application

**Figure 1.**
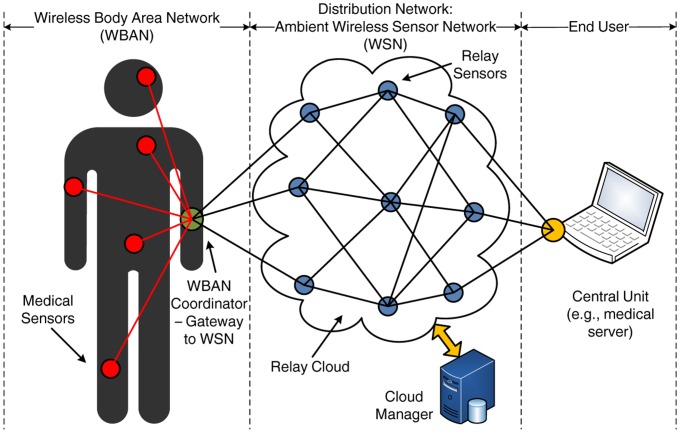
Framework of the end-to-end healthcare application.

We consider an end-to-end healthcare application, as illustrated in Figure 1. The system architecture can be decomposed into three parts:

1. The WBAN, formed by a number of medical sensors, is connected in a star topology to a central coordinator. The WBAN coordinator plays the role of a gateway between the WBAN and the distribution WSN, by collecting all the sensory data and forwarding them to the relay cloud. For simplicity, one WBAN, corresponding to a single patient, is considered in Figure 1; however, in a real case scenario, multiple WBANs can be supported.
2. The distribution WSN, composed of a number of ambient sensors in a mesh topology that act as relays. The relays have a static deployment and form a relay cloud. We explore two possible deployments: (i) there is no communication between the relays apart from the transmission of data (*i.e.*, no control information is exchanged); and (ii) all the relays in the cloud communicate through a dedicated high-speed connection (e.g., Ethernet link) with a coordinating entity, known as cloud manager.
3. The end user, which, without loss of generality, is a medical server that acts as a sink and receives all the medical data collected by the WBAN and forwarded by the relay cloud. The server can be accessed by the healthcare providers through an application interface. Clearly, several approaches of different complexity can be considered for this part, but they are beyond the scope of the paper.

Our work is mainly focused on the second part of the proposed architecture, namely the distribution WSN, which in our case, constitutes a relay cloud. The key idea is to study mechanisms for the transmission of data between the two ends of the network, *i.e.*, the WBAN coordinator and the end user. For convenience, from now on, we will refer to the WBAN coordinator as the source and to the end user as the destination. Nevertheless, this assumption is not restrictive, and more complex scenarios can also be supported, including the presence of multiple sources (*i.e.*, multiple patients) or the bidirectional flow of information (e.g., data sent from the healthcare provider to the patient).

### 2.2. System Model

**Figure 2.**
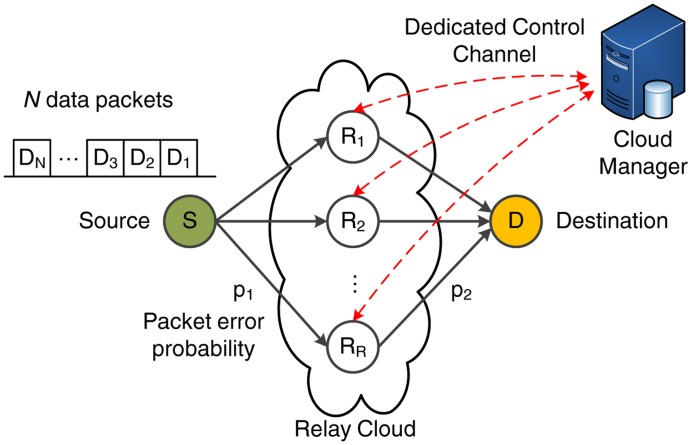
System model.

We consider the scenario shown in Figure 2, consisting of a source (S), a destination (D) and a cluster of R*_i_* relays (with *i* ∈ [1, *R*]) that form the relay cloud. In the remainder of this paper, we will use the notation source or S and destination or D interchangeably. In each transmission sequence, S transmits a set of *N* data packets of fixed length *L*. We assume that there is no direct connection between S and D, so all data transmitted by S must be forwarded to D in two hops through the relay cloud. Without loss of generality, a two-hop relay network is considered in order to identify the performance gains and weaknesses of the evaluated MAC schemes. The advantage of this setup is that it enables us to show, in a clear way, through analytical formulation and simulations, the need for the cloud-based coordination of the relays in order to fully exploit the benefits of RLNC schemes. The study of the two-hop case clearly illustrates the potential enhancements of the proposed centralized architecture, since the gain in more complex network topologies would be even more pronounced. In addition, the two-hop network can be conceived of as the basic building block to extend this work for a multi-hop case with minor modifications (such as defining a cluster head among the relays of each hop, responsible for the transmission of acknowledgment (ACK) frames when necessary).

The packet error probability of each wireless link follows a Bernoulli process with probability of failure *p* and success probability 1 − *p*. In terms of simplicity, we assume that the relay links are independent, but have similar average channel conditions, even though this assumption could be relaxed without significantly affecting the performance or the mathematical formulation of our proposed scheme. Hence, we define as *p*_1_ and *p*_2_ the packet error probability for the first and second hop, respectively. Clearly, a more sophisticated channel model that considers shadow fading and, possibly, correlation between the relay links should be adopted for the evaluation of real-life healthcare scenarios. Nevertheless, the scope of this paper is to emphasize how the proposed cloud-based MAC mechanism manages to alleviate the RLNC performance degradation when packets are lost due to link failures. For this task, the adopted Bernoulli-based erasure model is a valid assumption, since a more complex channel would not offer further insights with respect to the operation of the proposed schemes.

## 3. MAC Protocols for the Relay Network

In this section, we present three MAC protocols that handle the flow of information through the distribution network. First, BS-MAC is the baseline scheme that follows the IEEE 802.15.6 standard rules. Second, NC-MAC employs RLNC to enhance the system performance, but has several vulnerabilities in the presence of channel errors. Finally, CLNC-MAC is the proposed cloud-based solution that employs centralized control to fully exploit RLNC.

### 3.1. Baseline MAC (BS-MAC)

**Figure 3.**
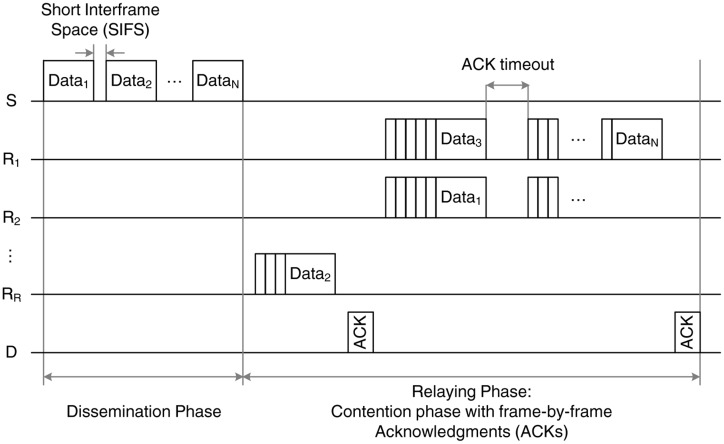
Example of the baseline medium access control (BS-MAC) transmission sequence.

The first scheme, BS-MAC, follows the IEEE 802.15.6 transmission rules with slight modifications to account for the two-hop transmission of a set of *N* packets. The transmission sequence of the BS-MAC, depicted in Figure 3, is divided in two phases:

- The dissemination phase, during which the source, S, transmits *N* data packets to the relays. These packets are transmitted in broadcast mode and, as a result, no acknowledgment (ACK) is issued by the relays. Due to the channel errors in the first link, every relay has a probability, 1 − *p*_1_, of correctly receiving each of the *N* data packets.
- The relaying phase, during which the relays forward the data packets to the destination, D. In this phase, the IEEE 802.15.6 rules for contention-based channel access are followed [3]. More specifically, all the relays sense the channel for a short inter-frame space (SIFS) period, and if they find it idle, they initiate a backoff counter selected randomly from a given contention window (CW). The counter is decreased in every idle slot, and when it reaches the value of zero, the relay attempts the data transmission. For every correctly received data packet, the destination transmits an ACK, and the relays remove the acknowledged packet from their buffer, thus avoiding the unnecessary retransmissions of the same data packet by different relays. In case of a collision or lack of ACK reception due to channel errors (occurring with probability *p*_2_), the backoff counter is reset accordingly, and a retransmission is attempted. We consider that the source enters into a sleep mode during the relaying phase, in order to reduce its energy consumption.

This scheme has some inherent weaknesses that stem from the lack of cooperation among the relays. In particular, two main issues can be identified:

1. The lack of ACKs in the dissemination phase. A necessary condition for the successful delivery of the data packets at the destination is to ensure that all *N* packets transmitted by the source are received by the relay cloud during the dissemination phase. In other words, each data packet should be correctly received by at least one relay; otherwise, the destination will not be able to retrieve all the original information, regardless of the number of retransmissions in the relaying phase. However, introducing ACKs in the dissemination phase is not straightforward. Either sequential ACKs should be sent from each relay for every packet, leading to substantial overhead, or a contention scheme for the ACK transmission should be employed.
2. The lack of coordination in the relaying phase. During the relaying phase, it is likely to have multiple data collisions, especially in the presence of many relays in the network, since all relays try to forward their received data.

### 3.2. RLNC-Based MAC (NC-MAC)

**Figure 4.**
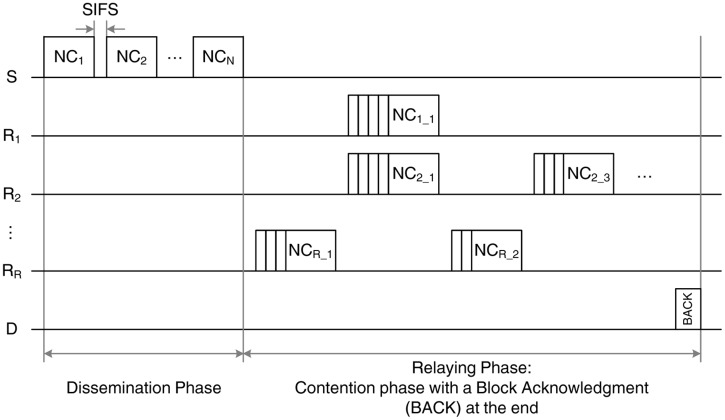
Example of the network coding (NC)-MAC transmission sequence.

The NC-MAC protocol aims to exploit diversity and limit the amount of exchanged control overhead by employing RLNC techniques. An example of the transmission sequence is given in Figure 4. As in the baseline scheme, two phases can be distinguished:

- The dissemination phase, in which S transmits *N* linear combinations of the *N* uncoded data packets (typically referred to as original packets). Each relay, R*_i_*, receives a subset, *M_i_* (0 ≤ *M_i_* ≤ *N*, ∀*i* ∈ [1, *R*]), of the transmitted packets, depending on the channel error probability, *p*_1_. This subset may be different for each relay, since the channel quality is independent for each link. The relays, then, create new linear combinations from the received packets and proceed to the next phase. It should be noted only up to *M_i_* linearly independent encoded packets can be generated by the *i*-th relay.
- The relaying phase, in which each relay forwards its encoded packets to the destination, D, following the IEEE 802.15.6 access rules. The destination is able to perform decoding and retrieve the original *N* data packets only after receiving *N* independent linear combinations that contain all the original information. After a successful decoding, the destination transmits a block ACK (BACK). Otherwise, if the destination is unable to retrieve the original information after a predefined timeout, the transmission sequence is terminated.

Clearly, the control overhead required for the application of the NC-based scheme is significantly reduced, since a single BACK frame is required, instead of the *N* ACKs employed by BS-MAC. However, there is a performance tradeoff, since, due to the lack of ACKs, the successful decoding of the original packets at the destination is not guaranteed. Moreover, the weaknesses identified in the case of BS-MAC still persist:

1. The lack of ACKs in the dissemination phase. The failure of the reception of a coded packet from the relay cloud could hinder the information decoding at the destination. As a result, the destination will not have sufficient linear combinations of the encoded data and will not be able to extract the original packets.
2. The lack of coordination in the relaying phase. Since there is no acknowledgment of the packet transmissions on a frame-by-frame basis, it is possible (although rare) to have multiple retransmissions of the same information (different encoded packets that are linearly dependent, thus not useful for the decoding process), hence wasting network resources.

## 3.3. Cloud-Assisted RLNC-Based MAC (CLNC-MAC)

In order to overcome the weaknesses of the previously described schemes, it is necessary to introduce some level of coordination between the relays. Accomplishing this in a distributed manner (e.g., by forming relay clusters and assigning cluster heads to coordinate the inter-relay exchange of information) can be a complicated task, especially if the network structure changes dynamically. Although the relay deployment is static, the source node (*i.e.*, the patient WBAN) may be moving. Hence, the set of relays that participate in the communication between S and D may vary with time.

**Figure 5.**
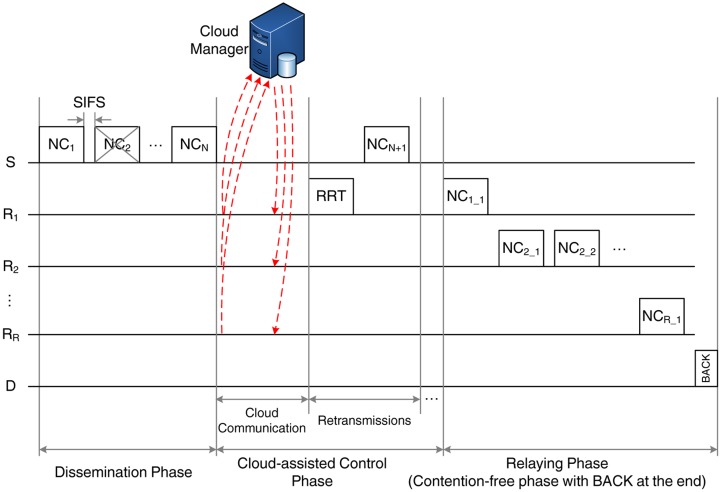
Example of the cloud-assisted random linear network coding (RLNC)-based (CLNC)-MAC transmission sequence.

Hence, we introduce CLNC-MAC, a cloud-assisted MAC protocol that improves the network performance through relay coordination. Our proposal adopts centralized control, assuming that the relays are able to exchange control information with a coordinating entity, the cloud manager, through a dedicated link. The transmission sequence of CLNC-MAC, shown in Figure 5, introduces some new features with respect to the reference schemes and can be divided into two phases:

- The dissemination phase, in which the reception of *N* original packets by the relay cloud is guaranteed. This phase consists of three parts:
  – The transmission phase, in which S transmits *N* linear combinations of the *N* original data packets to the relays, similar to NC-MAC.
  – The cloud-assisted control phase, in which the relay actions are coordinated with the help of the cloud manager. This phase has a variable duration and consists of two parts. In the first part, the relays simply exchange control information with the cloud manager, communicating the sequence number of the received packets. In the second part, there are two possible courses of action, depending on whether all the transmitted packets are available to the relay cloud: (i) If all *N* packets have been correctly received, the cloud manager establishes a transmission schedule for the relaying phase and notifies the relays accordingly (no retransmissions are required). (ii) While, if less than *N* packets have been received, then a retransmission phase takes place: one relay, designated by the cloud manager, transmits a request for retransmission (RRT) frame, asking for the retransmission of the missing packets by S. Since RLNC is employed, S does not have to retransmit the exact missing packets, but it simply needs to provide a sufficient number of linear independent combinations of the original packets. Once these packets are sent, the cloud communication phase takes place again and the process is repeated until all packets are correctly received by the cloud. Then, as in NC-MAC, the relays create new linear combinations from the received packets and proceed to the next phase.
- The *relaying phase*, in which the relays forward the encoded frames to the destination, D, according to the schedule issued by the cloud manager. The cloud coordination offers two important advantages. First, since relay transmissions follow a predefined schedule, collisions and backoff times are eliminated. Second, the number of frames that should be transmitted by each relay is determined by the schedule, thus avoiding the unnecessary retransmissions. Transmissions take place until D receives a sufficient number of copies to decode the original packets. Then, it terminates the relaying phase by transmitting a BACK.

**Figure 6.**
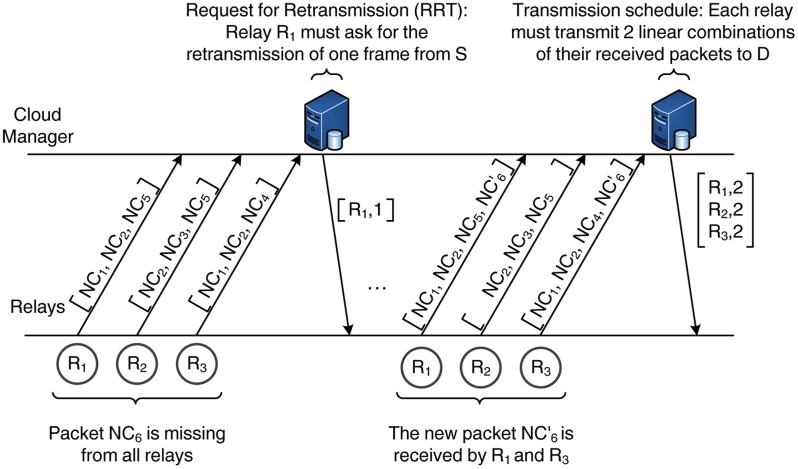
Information exchange between the relays and the cloud manager. RRT, request for retransmission.

Figure 6 provides a schematic description of the interaction between the relays and the cloud manager and the exchange of information during the cloud-assisted control phase. In this example, we consider that the source has transmitted six packets, NC_1_ – NC_6_, to a set of three relays. In the beginning of the cloud-assisted control phase, the relays transmit a vector with the sequence numbers of the received packets (but not the actual data). The cloud manager processes this information and observes that one packet (NC_6_) has not been received by any of the relays. Hence, it notifies a randomly selected relay (e.g., R_1_) to send an RRT and request for the transmission of one more encoded packet. Once S transmits the new packet ( $NC_{6}^{\prime }$), the relays notify the cloud manager again. In this case, the new packet has been received by two of the relays; therefore, the relay cloud possesses all the necessary information for the decoding of the source data (*i.e.*, there are six linear independent copies of the source data available to the relay cloud). As a next step, the cloud manager issues a scheduling vector that determines the transmission order of the relays and the number of packets to be transmitted. Here, we adopt a simple and fair approach by assigning the same number of packets to the relays whenever possible. Hence, in this example, each relay must transmit two linear combinations of the received packets.

Summarizing, Table 1 gives an overview of the main features of the three proposed schemes.

## 4. Mathematical Analysis of CLNC-MAC

In this section, we present an analytical model for the key performance metrics of CLNC-MAC. We have divided the analysis into two subsections. First, we describe the queuing model employed to approximate the protocols behavior. In particular, we model the cloud-assisted retransmissions and the relaying phase as absorbing Markov chain systems, since their duration is non-deterministic and depends on the channel errors. Then, in the second part, we provide definitions and closed-form expressions for the average throughput, the mean completion time and the energy efficiency of the CLNC-MAC protocol.

### 4.1. Queuing Model for CLNC-MAC

Three parameters are essential in order to obtain a theoretical estimation of the performance metrics of CLNC-MAC: (i) the expected number of RRT transmissions, **E**[*rrt*]; (ii) the expected number of retransmitted data packets, **E**[*rtx*]; and (iii) the expected number of relayed data packet transmissions, **E**[*rel*]. These parameters can be obtained by considering the different phases of the CLNC-MAC, as shown in Figure 5.

During the transmission part of the dissemination phase, the source transmits *N* data packets sequentially. Given that there are *R* relays and the packet error probability between the source and the relays is *p*_1_, the probability, *P_e_*, that a packet is not received by the relay cloud (*i.e.*, the probability that all the relays receive a given packet with errors) is $P_{e}=p_{1}^{R}$. Then, the probability, *P*(*n*), that *n* out of *N* packets are lost is given by:

(1) $$P(n)=\left(\begin{array}{l} N\\ n \end{array}\right){\left(P_{e}\right)}^{n}{\left(1-P_{e}\right)}^{N-n}$$

The cloud-assisted control phase has a variable duration, depending on the outcome of the transmission phase and the subsequent retransmissions. It is divided into two parts, the cloud communication and the retransmissions. No analysis is required for the cloud communication between the relays and the cloud manager, since it takes place through a dedicated and error-free link, and thus, we can assume that it has a deterministic duration.

As far as the retransmission phase is concerned, a number of retransmissions is requested to the source through an RRT frame, depending on the packets lost during the initial transmission (as expressed by Equation (1)). However, due to the channel errors, it is possible that some of the retransmitted frames are again lost by all relays. Hence, more retransmissions are requested through another RRT frame, and this recursive process is repeated, until all *N* packets become available to the relay cloud.

**Figure 7.**
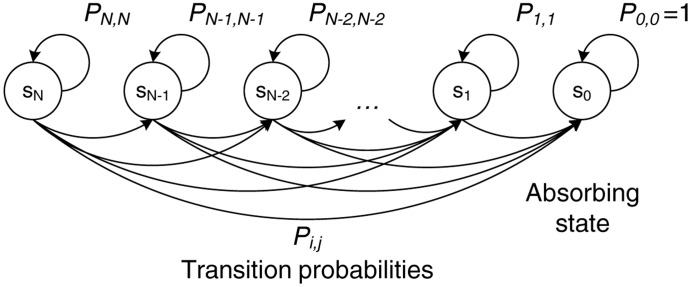
Absorbing Markov chain model for the retransmission phase.

This process can be modeled as a Markov chain with one absorbing state, as illustrated in Figure 7. Each state of the chain, denoted by *s_i_*, represents the number of retransmissions, *i*, with *i* ∈ [0, *N*], requested in the RRT frame. After each retransmission round, the system may remain at the same state with probability *P_i,i_* if none of the retransmitted packets is received by the relay cloud or move to another state, *s_j_*, (where *j* < *i*) with probability *P_i,j_*. When the absorbing state, *s*_0_, is reached and no more retransmissions are required, the process is terminated (*i.e.*, the system remains in that state with *P*_0,0_ = 1).

Therefore, the transition probabilities, *P_i,j_*, for moving from state *i* to state *j* can be expressed as:

(2) $$P_{i,j}=\{\begin{array}{ll} 0 & ,j>i\\ P_{e}^{i} & ,j=i\\ \left(\begin{array}{l} i\\ j \end{array}\right){\left(P_{e}\right)}^{j}{\left(1-P_{e}\right)}^{i-j} & ,j<i \end{array}$$

We then form the canonical form of the transition matrix, by ordering first the transition probabilities of the transient states (*i.e.*, *s_N_* to *s*_1_):

(3) $$P_{tr}=\left[\begin{array}{cc} \begin{array}{cccc} P_{N,N} & P_{N-1,N-1} & \cdots & P_{N,1}\\ 0 & P_{N-1,N-1} & \cdots & P_{N-1,1}\\ \vdots & \vdots & \ddots & \vdots \\ 0 & 0 & 0 & P_{1,1} \end{array} & \begin{array}{c} P_{N,0}\\ P_{N-1,0}\\ \vdots \\ P_{1,0} \end{array}\\ \begin{array}{cccc} 0 & \;0 & \;0 & \;0 \end{array} & 1 \end{array}\right]=\left[\begin{array}{cc} \text{Q} & \text{R}\\ 0 & \text{I} \end{array}\right]$$

where **Q** is an *N* × *N* nonzero matrix with the transition probabilities among the transient states, **R** is an *N* × 1 nonzero matrix with the transition probabilities from the transient states to the absorbing state, *s*_0_, 0 is an 1 × *N* zero matrix and **I** is an 1 × 1 identity matrix.

For an absorbing Markov chain, it is proven that the matrix, **I** − **Q**, is invertible, and its inverse, **N_p_**, is called the fundamental matrix of the chain:

(4) $${\text{N}}_{\text{p}}={\left(\text{I}-\text{Q}\right)}^{-1}$$

The element, *n_Pij_*, of the fundamental matrix, **N_p_**, expresses the expected number of times that the process is in the transient state, *s_j_*. Hence, the sum of all the entries in the *i*-th row of **N_p_**, denoted by **E**[*t_i_*], gives the expected number of times in any of the transient states for a given starting state, *s_i_*, or, equivalently, the expected number of steps before being absorbed by state *s*_0_:

(5) $$\text{E}[t_{i}]=\sum_{j=1}^{N}n_{Pij}$$

In terms of the system performance, **E**[*t_i_*] corresponds to the number of retransmission rounds (initiated by an RRT transmission) when *i* packets are lost during the transmission phase (*i.e.*, when the starting state is *s_i_*). The expected number of RRT transmissions, **E**[*rrt*], can be obtained by combining Equations (1) and (5):

(6) $$\text{E}[\text{rrt}]=\sum_{j=1}^{N}P(i)\text{E}[t_{i}]$$

In a similar way, the expected number of retransmitted packets, **E**[*rtx_i_*] for a given starting state, *s_i_*, is given by:

(7) $$\text{E}[{\text{rtx}}_{i}]=\sum_{j=1}^{N}jn_{Pij}$$

and the expected number of retransmitted data packets, **E**[*rtx*], is calculated as:

(8) $$\text{E}[\text{rtx}]=\sum_{i=1}^{N}P(i)\text{E}[{\text{rtx}}_{i}]$$

**Figure 8.**
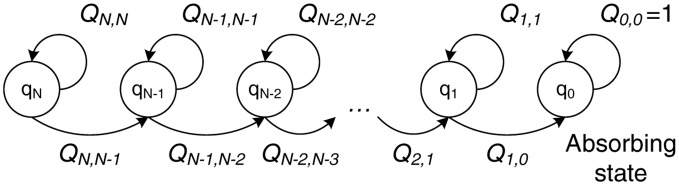
Absorbing Markov chain model for the relaying phase.

Finally, in order to calculate the third parameter, *i.e.*, the expected number of relayed data packet transmissions, **E**[*rel*], we need to consider a queuing model for the relaying phase. During the relaying phase, the destination must correctly receive *N* packets from the relay cloud, given that the link between the relays and the destination has a channel error probability equal to *p*_2_. We model this process by considering the absorbing Markov chain depicted in Figure 8.

Each state, *q_i_*, represents the number of data packets, *i* (with *i* ∈ [0, *N*]), required by the destination. Whenever a packet is transmitted by the relays, the system may either remain at the same state, *q_i_*, with probability *Q_i,i_* if the packet is received with errors, or move to the immediately lower state, *q_i_*_−1_, with probability *Q_i,i_*_−1_ if the packet is correctly received. When all *N* packets are received, the system reaches the absorbing state, *q*_0_, and remains there with probability *Q*_0,0_ = 1.

Hence, the transition probabilities, *Q_i,j_*, for moving from state *i* to state *j* can be expressed as:

(9) $$Q_{i,j}=\{\begin{array}{ll} 1 & ,j=i=0\\ p_{2} & ,j=i,i\neq 0\\ 1-p_{2} & ,j=i-1\\ 0 & ,\text{otherwise} \end{array}$$

Following the same line of thought, we form the canonical form of the transition matrix as:

(10) $$Q_{tr}=\left[\begin{array}{cc} \begin{array}{ccccc} Q_{N,N} & Q_{N-1,N-1} & 0 & \cdots & 0\\ 0 & Q_{N-1,N-1} & Q_{N-1,N-2} & \cdots & 0\\ \vdots & \vdots & \vdots & \ddots & \vdots \\ 0 & 0 & 0 & \cdots & Q_{1,1} \end{array} & \begin{array}{c} 0\\ 0\\ \cdots \\ Q_{1,0} \end{array}\\ \begin{array}{ccccc} 0 & \;0 & \;0 & \;\cdots & \;0 \end{array} & \begin{array}{c} 1 \end{array} \end{array}\right]=\left[\begin{array}{cc} \text{Q} & \text{R}\\ 0 & \text{I} \end{array}\right]$$

We then calculate the fundamental matrix of the chain as **N_q_** = (**I** − **Q**)^−1^. As in Equation (5), the expected number of steps before being absorbed, spent in any of the transient states for a given starting state, *q_i_*, is given by the sum of all the entries in the *i*-th row of N_q_. In this case, however, *q_N_* is always the starting state of the system, since at the beginning of the relaying phase, *N* packets are required by the destination. Hence, the expected number of data transmissions by the relays, **E**[*rel*], can be calculated as:

(11) $$\text{E}\left[\text{rel}\right]=\sum_{j=1}^{N}n_{QN_{j}}$$

where *n_QNj_* are the entries of the fundamental matrix, **N_q_**, for the *N*-th row.

### 4.2. Performance Metrics for CLNC-MAC

Having estimated the three parameters, **E**[*rrt*], **E**[*rtx*] and **E**[*rel*], in Equations (6), (8) and (11), respectively, we can proceed to the evaluation of the performance metrics.

Throughput *S* is defined as the average data transmission rate of the system and can be calculated as the expected number of the total correctly received data bits, **E**[*x*], at the destination during a transmission sequence, divided by the expected time duration, **E**[*t*], of the transmission sequence, counting from their transmission by the source until the correct reception by the destination:

(12) $$\text{E}\left[S\right]=\frac{\text{E}\left[x\right]}{\text{E}\left[t\right]}$$

In CLNC-MAC, the reception of all the transmitted packets is guaranteed, given that a sufficient number of retransmissions takes place. Hence, if *N* data packets of *L* bytes are transmitted by the source, then **E**[*x*] is calculated as:

(13) E[x]=8LN

Based on Figure 5, the expected duration of the transmission sequence, which constitutes the second important performance metric, can be estimated as:

(14) $$\text{E}[t]=\text{E}[t_{tx}]+\text{E}[t_{\text{cloud}}]+\text{E}[t_{\text{retx}}]+\text{E}[t_{\text{relays}}]$$

where **E**[*t_tx_*], **E**[*t_cloud_*], **E**[*t_retx_*] and **E**[*t_relays_*] denote the expected duration of the transmission phase, the cloud communication, the retransmissions and the relaying phase, respectively.

To calculate these values, we define as *t_DATA_*, *t_RRT_*, *t_BACK_* the transmission time of the data, the RRT and the BACK packets, respectively. These times are deterministic, depending on the packet size and the transmission data rate of each frame. We also define as *t_SIFS_* the SIFStime inserted between consecutive packet transmissions. The different terms of Equation (14) are calculated next.

The duration of the transmission phase is also deterministic, since it involves the time required for the transmission of *N* data packets by the source:

(15) $$\text{E}[t_{tx}]=Nt_{\text{DATA}}+(N-1)t_{\text{SIFS}}$$

The cloud communication between the relays and the cloud manager through the dedicated link has a constant duration, *t_cl_*, that is considered known. However, the number of cloud communication sessions is not fixed, since at least one session takes place after the dissemination phase and subsequent sessions may be required, depending on the number of retransmission rounds, **E**[*rrt*], given by Equation (6). Hence,

(16) $$\text{E}[t_{\text{cloud}}]=(1+E[\text{rrt}])t_{cl}$$

The retransmission phase consists of **E**[*rrt*] retransmission rounds, during which an average of [*rtx*] data packets, given by Equation (8), are retransmitted by the source. As a result, the term, **E**[*t_retx_*], is calculated as:

(17) $$\text{E}[t_{\text{retx}}]=\text{E}[\text{rrt}](t_{\text{RRT}}+t_{\text{SIFS}})+\text{E}[\text{rtx}](t_{\text{DATA}}+t_{\text{SIFS}})$$

Finally, the relaying phase involves the transmission of **E**[*rel*] data packets by the relays, as calculated by Equation (11), and the transmission of the BACK by the destination. Therefore,

(18) $$\text{E}[t_{\text{relays}}]=\text{E}[\text{rel}](t_{\text{DATA}}+t_{\text{SIFS}})+t_{\text{BACK}}+t_{\text{SIFS}}$$

The third metric of interest is the energy efficiency, **E**[*η*], defined as the expected number of the total correctly received useful data bits, **E**[*x*], at the destination during a transmission sequence (given by Equation (13)), divided by the total energy, **E**[*E_t_*], consumed by the system during the same time.

(19) $$\text{E}\left[\eta \right]=\frac{\text{E}\left[x\right]}{\text{E}\left[E_{t}\right]}$$

Taking into account the protocol operation, the total energy consumption of the system is:

(20) $$\text{E}[E_{t}]=\text{E}[E_{S}]+\text{E}[E_{D}]+\text{E}[E_{R}]$$

where **E**[*E_S_*], **E**[*E_D_*] and **E**[*E_R_*] denote the average energy consumed by the source, the destination and the relay cloud, respectively. We assume that all devices have the same power consumption levels, namely *P_tx_*, *P_rx_*, *P_idle_* and *P_sleep_*, for transmission, reception, idle and sleep mode, respectively.

Based on the transmission sequence of the CLNC-MAC, demonstrated in Figure 5, the energy consumption of the source is:

(21) $$\begin{array}{ll} \text{E}[E_{S}] & =P_{tx}\left(N+\text{E}[\text{rtx}]\right)t_{\text{DATA}}\\ & +P_{rx}\text{E}\left[\text{rrt}\right]t_{\text{RRT}}\\ & +P_{\text{idle}}\left(\left(\left(N-1)+\text{E}[\text{rrt}]+\text{E}[\text{rtx}\right]\right)t_{\text{SIFS}}+\text{E}[t_{\text{cloud}}]\right)\\ & +P_{\text{sleep}}\left(\text{E}[\text{rel}]\left(t_{\text{SIFS}}+t_{\text{DATA}}\right)+t_{\text{BACK}}+t_{\text{SIFS}}\right) \end{array}$$

since the source transmits *N* + **E**[rtx] data packets, receives the RRT frames, sleeps during the relaying phase and stays idle for the remaining transmission sequence duration.

On the other hand, the energy consumption of the destination is:

(22) $$\begin{array}{ll} \text{E}\left[E_{D}\right] & =P_{tx}t_{\text{BACK}}\\ & +P_{rx}\left(\text{E}[\text{rrt}]t_{\text{RRT}}+\text{E}[\text{rel}]t_{\text{DATA}}\right)\\ & +P_{\text{idle}}\left(\left(\left(N-1)+\text{E}[\text{rrt}]+\text{E}[\text{rtx}\right]\right)t_{\text{SIFS}}+\left(N+\text{E}[\text{rtx}]\right)t_{\text{DATA}}+\text{E}[t_{\text{cloud}}]\right) \end{array}$$

given that the destination transmits the BACK frame, receives the relayed data and the RRT frames and stays idle for the remaining transmission sequence duration (no sleep mode is considered in this case).

Finally, the total energy consumption of the *R* relays forming the cloud can be estimated as:

(23) $$\begin{array}{ll} \text{E}\left[E_{R}\right] & =P_{tx}\left(\text{E}[\text{rrt}]t_{\text{RRT}}+\text{E}[\text{rel}]t_{\text{data}}\right)\\ & +P_{rx}\left(R\left((N+\text{E}[\text{rtx}])t_{\text{DATA}}+t_{\text{BACK}}\right)+(R-1)\left(\text{E}[\text{rrt}]t_{\text{RRT}}+E[\text{rel}]t_{\text{DATA}}\right)\right)\\ & +P_{\text{idle}}\left(R\left(\left(N-1)+\text{E}[\text{rrt}]+\text{E}[\text{rtx}]+\text{E}[\text{rel}]+1+\text{E}[t_{\text{cloud}}\right]\right)t_{\text{SIFS}}]\right) \end{array}$$

Since only one relay at a time transmits the RRT frames and the relayed data, all relays receive the transmissions and the retransmissions by the source, as well as the BACK packet by the destination, *R*−1 relays receive the RRT frame and the relayed data packets, and all relays stay idle for the remaining transmission sequence duration.

Summarizing, we have derived closed-form expressions through queuing analysis for the key performance metrics of CLNC-MAC, namely the average throughput, the mean completion time and the energy efficiency. Even though this model reflects the protocol's operation for the considered two-hop scenario, the queuing model can be extended for the multi-hop case (*i.e.*, each hop would consist of another cloud-assisted control and relaying phase). Of course, some modifications in the protocol's rules would be required for multi-hop operation, but the queuing mechanism would basically be the same. Finally, it should be noted that the proposed model is not limited to the Bernoulli error model. In fact, any channel model that allows the calculation of the average packet error rate could be directly applied.

## 5. Performance Evaluation

In this section, we evaluate the performance of the three MAC schemes described in Section 3. A C++ simulator has been developed for the performance evaluation of the proposed schemes. The code executes all the steps of the transmission sequence of the proposed schemes, incorporating the channel model and applying, when necessary, the contention rules defined in the IEEE 802.15.6 standard. The simulation setup is given in Section 5.1, while the obtained results are presented and discussed in Section 5.2.

### 5.1. Simulation Setup

In each transmission sequence, a set of *N* = 10 data packets has been generated by the source, with a packet payload size of *L* = 100 bytes. When RLNC is employed (*i.e.*, in the case of NC-MAC and CLNC-MAC), a Galois field of 2^8^ has been considered, since it has been proven to be sufficient for linear independence among the packets [15]. The specific field implies an overhead of eight bits for each packet in the linear combination. The control packets (RTT and BACK) only consist of the MAC header with no payload. Regarding the power levels, we assume that all nodes consume 40 mW in transmission mode (*i.e.*, *P_tx_* = 40 mW), and they have the same consumption *P_rx_* = *P_idle_* = 20 mW in reception and idle mode, respectively. The power consumption in sleep mode is almost negligible, i.e., *P_sleep_* = 1 mW.

The Physical (PHY) and MAC layer parameter values have been chosen, such that they are compatible with the IEEE 802.15.6 standard for WBANs [3]. In particular, the narrowband PHY specification at 2.4 GHz has been considered, with a rate of 485.7 kbps for the data packet transmission and the lowest rate of 121.4 kbps for the control packets. We assume that the transmission of the control packets is error-free, whereas the data packets have an error probability of *p*_1_ and *p*_2_ in the first and second hop, respectively. The packet duration has been calculated according to the standard guidelines (Equation (77) of [3]). Finally, for BS-MAC and NC-MAC, the CW bounds for the selection of the backoff counter have been set to [16, 64]. The simulation parameters are summarized in Table 2.

### 5.2. Results

**Figure 9.**
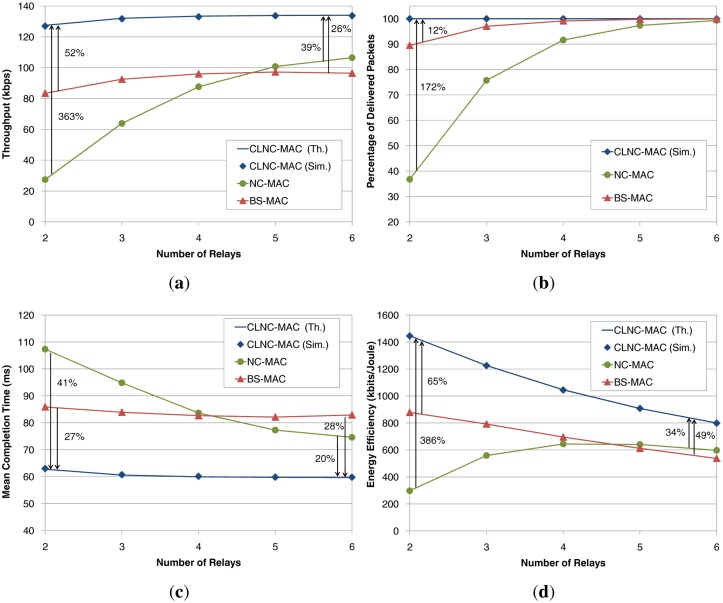
Performance evaluation through the analytical model and simulations (depicted by Th. and Sim., respectively) as a function of the number of relays, for *p*_1_ = *p*_2_ = 0.3. (**a**) Throughput; (**b**) percentage of successfully delivered packets; (**c**) mean data delay; (**d**) energy efficiency.

The first set of plots in Figure 9 shows the performance of the three schemes, BS-MAC, NC-MAC and CLNC-MAC, as a function of the number of relays, *R*. In this setup, the packet error probability for all links has been set to *p*_1_ = *p*_2_ = 0.3. In addition, the time required for the communication between the relays and the cloud manager has been considered negligible, *i.e.*, *t_cl_* = 0. As an overall observation, there is a very close match between the theoretical values (solid line) and the simulation results (diamond marker) for the CLNC-MAC, thus proving the validity of the proposed analytical model.

Figure 9a shows the throughput performance of the three schemes. In the case of BS-MAC, throughput gradually increases with the number of relays and reaches a maximum value of approximately 97 kbps. NC-MAC slightly improves throughput when more than five relays are present, but yields a very low throughput for a small number of relays, due to the packet errors introduced by the channel in both links (source to the relays and relays to the destination). As mentioned in Section 3.2, the lack of ACKs in the dissemination phase, along with the lack of coordination in the relaying phase, have a devastating effect on the performance of NC-MAC. By comparing NC-MAC to the BS-MAC, it can be said that the application of RLNC actually deteriorates the system performance for a small number of relays. On the other hand, CLNC-MAC has a superior throughput performance compared to both reference schemes, even for a very small number of relays, since it ensures the reception of all data by the relay cloud and their contention-free delivery to the destination. In particular, a throughput gain of 52% and 363% is achieved with respect to the BS-MAC and the NC-MAC, respectively.

Figure 9b shows the percentage of delivered packets at the destination with respect to the transmitted packets by the source. In BS-MAC, 90% of the packets are delivered for *R* = 2 relays, but eventually, 100% is achieved for *R* = 6. The lost packets are mainly due to the lack of ACKs in the dissemination phase. As the number of relays grows, the probability of having each data packet received by at least one relay increases. Thus, the destination has a higher chance of receiving all the transmitted data packets. In the case of NC-MAC, the lack of ACKs has a much stronger impact, causing a remarkably low percentage of delivered packets for a small number of relays (less than 40% for *R* = 2). Since the transmitted packets by the source are linear combinations of the original data packets, even if only one out of the *N* packets is not correctly received by any of the relays during the dissemination phase, decoding at the destination cannot be performed, and none of the original packets can be retrieved. Finally, CLNC-MAC guarantees the successful delivery of all transmitted packets, regardless of the number of relays. Hence, it can be claimed that, in order to exploit the capabilities of RLNC techniques, the coordination among the relays, provided in the case of CLNC-MAC through the cloud manager, plays a crucial role, yielding a gain of up to 172% over NC-MAC for *R* = 2.

Figure 9c depicts the mean data delay for the three schemes, which corresponds to the average time to deliver the total information to the destination through the relay cloud. In the case of a few relays in the network, BS-MAC outperforms NC-MAC in terms of delay, but as the number of relays increases, NC-MAC achieves a better completion time for the end-to-end data transmission. However, the proposed CLNC-MAC exploits RLNC in a more efficient way, thus offering a completion time reduction of 28% and 41% compared to BS-MAC and NC-MAC, respectively.

Finally, as far as the energy efficiency is concerned (Figure 9d), CLNC-MAC shows the best performance, with gains of 65% and 386% with respect to BS-MAC and NC-MAC, respectively. This enhancement is obtained since CLNC-MAC guarantees the delivery of all transmitted packets (*i.e.*, offers a higher number of received bits at the destination) and reduces the time, and therefore, the energy, wasted in collisions due to the contention-free relaying phase. It is also worth noting that, as the number of relays grows, the energy efficiency decreases as expected, since CLNC-MAC guarantees the throughput and the packet delivery, independent of the number of the relays in the network.

**Figure 10.**
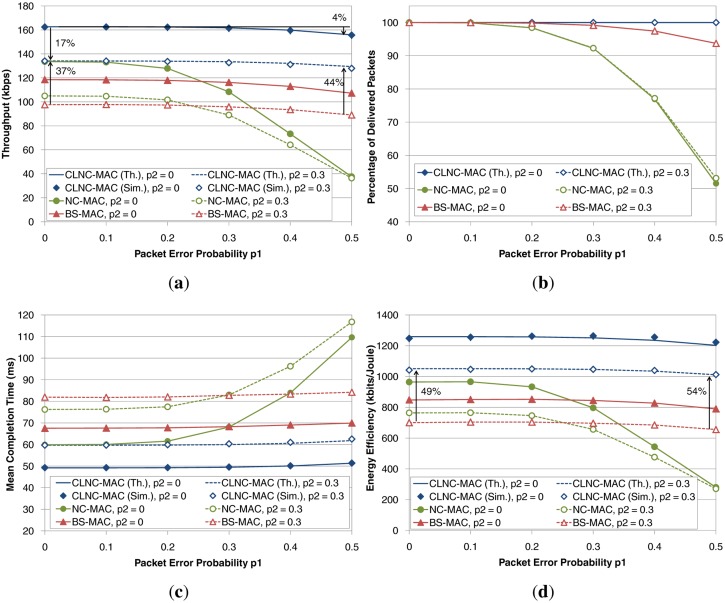
Performance evaluation as a function of the packet error probability between source and relays *p*_1_, for *R* = 4 relays. (**a**) Throughput; (**b**) percentage of successfully delivered packets; (**c**) mean data delay; (**d**) energy efficiency.

The second set of plots in Figure 10 shows the performance of the three schemes as a function of the packet error probability in the first hop (*i.e.*, between the source and the relays), *p*_1_, for a fixed number of relays equal to *R* = 4. Two different values for the packer error probability between the relays and the destination have been considered, namely *p*_2_ = 0 (solid lines) and *p*_2_ = 0.3 (dashed lines). In general, the first link is more likely to suffer from channel errors, since the source corresponds to a potentially mobile patient, whereas the second link is more predictable given the static deployment of the relay nodes and the destination. Again, it has been assumed that the cloud communication is instantaneous (*t_cl_* = 0).

In terms of throughput, depicted in Figure 10a, CLNC-MAC achieves the best performance. As *p*_1_ increases, throughput slightly drops, since more retransmission rounds are required to ensure the reception of data packets by the relay cloud. However, thanks to the diversity in reception obtained by the presence of *R* = 4 relays, the throughput degradation is very low (no more than 4% for *p*_1_ = 0.5 and *p*_2_ = 0, with respect to the error-free case of *p*_1_ = 0). On the other hand, errors in the second link have a stronger impact on throughput, due to the lack of reception diversity (*i.e.*, there is only a single destination in this scenario), which results in a 17% decrease for *p*_1_ = 0. BS-MAC shows a similar behavior as CLNC-MAC, but attains lower values of throughput (a gain from 37%–44% is obtained by CLNC-MAC for *p*_2_ = 0.3). Finally, for low values of *p*_1_, NC-MAC outperforms BS-MAC, due to the RLNC gain. However, throughput drops as the error probability grows, since many packets are lost during the first phase, thus reducing the probability of successful decoding at the destination.

The impact of the lost packets can also be verified by observing the percentage of delivered packets, plotted in Figure 10b. All schemes achieve the delivery of all packets when the introduced channel errors are very low, but only CLNC-MAC maintains this percentage at 100%, regardless of the error probability. Another interesting observation is that the error probability of the second link, *p*_2_, hardly affects the percentage of delivered packets (*i.e.*, the dashed and solid lines in the plot practically overlap). As a conclusion, it can be claimed that errors in the first phase determine whether the relay cloud will have all the necessary information to forward to the destination, whereas errors in the relaying phase require a higher number of retransmission, leading to a worse performance in terms of throughput, delay and energy efficiency, but without affecting the percentage of delivered packets.

With respect to the mean completion time, plotted in Figure 10c, BS-MAC has a slightly worse performance than NC-MAC for low values of *p*_1_. As *p*_1_ grows, the delay of BS-MAC remains constant, while the completion time for NC-MAC is increasing almost exponentially for error probabilities greater than 0.3 (*i.e.*, *p*_1_ > 0.3). On the other hand, CLNC-MAC is able to provide a constant completion time and lower compared to the two reference schemes, regardless of the packet error rate in the first hop. The introduction of errors in the second hop (*i.e.*, the case of *p*_2_ = 0.3) increases the delay of all schemes, without affecting the supremacy of CLNC-MAC compared to BS-MAC and NC-MAC.

The last plot (Figure 10d) shows the energy efficiency performance of the three schemes. In general, energy efficiency follows the same trend as throughput, with CLNC-MAC achieving the highest values compared to the other schemes. For reference, the energy efficiency gain of CLNC-MAC over BS-MAC ranges from 48% for *p*_1_ = 0 to 54% for *p*_1_ = 0.5, while the energy gain is even higher compared to the NC-MAC, whose performance deteriorates considerably for relatively high packet error probabilities.

**Figure 11.**
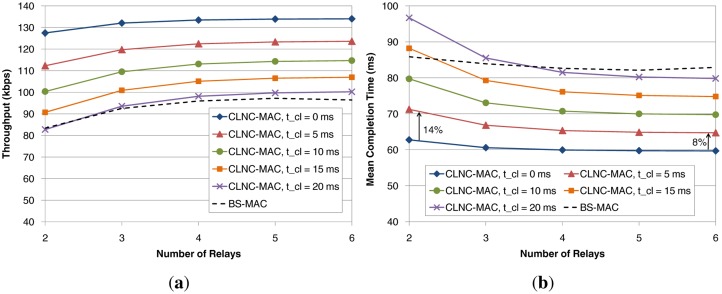
Impact of the cloud communication time on the performance of CLNC-MAC. (**a**) Throughput; (**b**) mean data delay.

Finally, the impact of the cloud communication time on the performance of CLNC-MAC as a function of the number of relays has been evaluated in Figure 11. Different values of *t_cl_* ranging from 0 ms (negligible delay) up to 20 ms have been considered, and the performance of BS-MAC (dashed line) has also been plotted as a reference. In this figure, only throughput and mean completion time are depicted, since the percentage of successfully delivered packets is not affected by *t_cl_* (it remains 100%), and energy efficiency shows a similar behavior as throughput.

Without doubt, smaller values of *t_cl_* yield a better performance. In terms of throughput (plot Figure 11a), it can be observed that as *t_cl_* grows, the CLNC-MAC performance gradually approaches BS-MAC, and both protocols achieve practically the same throughput for *t_cl_* = 20 ms. In terms of delay (plot Figure 11b), CLNC-MAC achieves a lower completion time in most cases, and more specifically, there is a margin of 20 ms before the two schemes achieve similar performance.

Hence, Figure 11 shows the latency requirements that must be satisfied by the cloud communication technology, in order to guarantee the efficient performance of CLNC-MAC. In any case, these requirements are not very demanding, considering that: (i) the link between the relays and the cloud manager is likely to be based on a high-speed cabled technology (e.g., Ethernet) with low round trip times; (ii) the data exchanged between the relays and the cloud manager is minimal, of the order of a few bytes; and (iii) the processing time is also negligible, since the cloud manager has high processing power and the complexity of the implemented algorithms is very low.

## 6. Conclusions

In this paper, we have investigated different MAC mechanisms in a network of relays, employed as the distribution network in an end-to-end healthcare application. Focusing on a two-hop scenario, we have studied the impact of channel errors on an IEEE 802.15.6-based scheme, which become even more detrimental when RLNC techniques are applied. As a solution, we have designed a cloud-assisted MAC protocol that guarantees the delivery of all data packets to the destination by enabling the coordination among the relays, thus exploiting the potential benefits of RLNC techniques. The proposed architecture can be applied in scenarios where the network of relays is connected to the cloud and can exchange information with the cloud manager with relatively low latency. For instance, an ambient WSN deployment with backhaul connectivity in a hospital ward would greatly benefit from the coordination offered by the cloud-based architecture.

The performance evaluation, through both mathematical analysis and simulations, has shown promising results. Significant enhancements have been achieved in terms of average throughput and energy efficiency, whereas there is a very slight increase in the delay performance, due to the application of RLNC. The presented results emphasize the need for centralized coordination in order to yield the maximum potential benefits of RLNC in one-way multi-hop networks in the presence of channel errors. Clearly, the cloud-based coordination gives the optimal solution for the reliable delivery of the transmitted packets, with some cost on the throughput and delay in cases where the cloud communication suffers from high latencies. In those cases, it would be interesting to explore performance tradeoffs depending on the desired application goal (e.g., the reliable delivery of all packets *versus* reduced completion time).

The work presented in this paper opens many new interesting lines of investigation. First, CLNC-MAC could be easily applied to a more complex topology with multiple sources and relay hops. The communication between the relays and the cloud manager can be further exploited to design more advanced scheduling and routing schemes, which take into consideration parameters, such as the channel quality of the relay links, the battery level of the devices or the fairness criteria. More realistic channel models for the link error probability can also be considered, encouraging the design of opportunistic scheduling and routing schemes. Finally, a very interesting and challenging task would be a testbed implementation of the cloud-based architecture, since it would enable us to improve practical issues and enhance the feasibility of the proposed schemes.

## Figures and Tables

**Table 1.** Comparison of the three MAC schemes.

**MAC-Scheme**	**BS-MAC**	**NC-MAC**	**CLNC-MAC**
Strengths	Simple and distributed	RLNC reduces overhead and exploits relay diversity	Guaranteed packet delivery, collision-free relaying
Weaknesses	Increased overhead (ACKs); delivery not guaranteed	Significant degradation from channel errors (decoding failure)	Increased complexity; cloud communication latency

Relay Coordination	None	None	Cloud-based
Complexity	Very low	Low (RLNC encoding/decoding)	Medium (RLNC and cloud processing)
Relaying Phase	Contention-based	Contention-based	Contention-free
Packet Delivery Guarantees	None	None	100% delivery

**Table 2.** Simulation Parameters.

**Parameters**	**Values**	**Parameters**	**Values**
*N* (packets)	10	PHY Preamble (bits)	90
*L* (bytes)	100	PHY Header (bits)	31
*R* (relay number)	2 to 6	[*CW_min_*, *CW_max_*]	[16, 64]
Galois Field	2^8^	*t_SIFS_* (ms)	0.075
MAC Header (bytes)	7	Data Tx Rate (kbps)	485.7
FCS (bytes)	2	Control Tx Rate (kbps)	121.4
*P_tx_* (mW)	40	*P_rx_* (mW)	20
*P_idle_* (mW)	20	*P_sleep_* (mW)	1
